# Beyond opioids: FDA-approved suzetrigine offers hope for acute pain management

**DOI:** 10.1097/MS9.0000000000003462

**Published:** 2025-06-10

**Authors:** Aleeza Khan, Maryam Irshad, Zara Javed, Zahra Naz, Mahnoor Fatima

**Affiliations:** a Department of Medicine, Nishtar Medical College, Nishtar Medical University, Multan, Pakistan; b Department of Medicine, Shaheed Ziaur Rahman Medical College, Rajshahi Medical University, Bogura, Bangladesh

The opioid epidemic is an ongoing public health crisis that has affected millions of people all over the world over the past two decades. It was officially declared a Public Health Emergency by the U.S. Department of Health and Human Services in 2017 and unfortunately has claimed the lives of hundreds of thousands of Americans. A recent WHO pharmacovigilance analysis reveals a substantial burden of opioid-related adverse drug reactions in high-income countries such as the United States, United Kingdom, Canada, and Australia, while suggesting widespread underreporting in low and middle-income regions[1]. The consequences of the opioid crisis are severe. Opioid-related mortality has increased significantly in recent years, with region-specific death tolls revealing the widespread nature of the epidemic. In the United States, the CDC’s State Unintentional Drug Overdose Reporting System recorded 51 435 overdose deaths in 2022[2]. On a global scale, approximately 60 million people are affected by addiction to prescription opioids, including hydrocodone, methadone, meperidine, oxycodone, and fentanyl. More than 100 000 individuals die each year from opioid overdoses worldwide. These figures reflect a pressing need for safer, non-addictive alternatives to address acute pain without contributing to the cycle of dependence and harm[3].

Opioids are a class of drugs primarily used to manage moderate to severe pain. They can be derived naturally from the opium poppy plant or synthesized in laboratories. These agents exert their analgesic effects by binding to opioid receptors located on nerve cells in the brain, spinal cord, gastrointestinal tract, and other organs, thereby blocking pain signals. Over the past several decades, opioids have been widely utilized not only as surgical analgesics but also in the treatment of cancer-related pain, diarrhea, tooth decay, and even as adjuncts for insomnia. They have also been misused for recreational purposes[4]. In 2023, approximately 125 million opioid prescriptions were dispensed to American patients, with significant variation across different states. Clinical guidelines recommend prescribing the lowest effective dose for the shortest duration possible to reduce the risk of harm. Despite these recommendations, nearly 8.6 million Americans aged 12 and older reported misusing prescription opioids in 2022. This widespread misuse is contributing to rising rates of addiction and dependency, highlighting the urgent need for more responsible prescribing practices and stronger public health interventions[5].

Amidst the ongoing challenges of the opioid crisis, the recent approval of Suzetrigine by the US Food and Drug Administration offers hope as a groundbreaking non-opioid analgesic, designed to treat short-term pain in adults. Suzetrigine is a highly potent, selective NaV1.8 inhibitor with a novel mechanism of action and no addictive potential. It specifically targets NaV1.8 in the peripheral nervous system rather than the brain and binds to a unique site with an allosteric mechanism distinct from local anesthetics. By inhibiting pain-signaling nerves, it prevents the transmission of painful sensations to the brain. Suzetrigine does not affect other CNS targets, and *in vivo* studies in monkeys and rats showed no CNS activity or addictive effects. Clinical trials involving over 2400 individuals also found no evidence of abuse or addiction[6].

Compared to opioids, Suzetrigine offers several advantages. It does not exhibit the same level of addictive potential, reducing the risk of dependence and overdose. Furthermore, Suzetrigine’s side effect profile is more favorable, with fewer reports of nausea, vomiting, and sedation. These benefits make Suzetrigine an attractive alternative for acute pain management, particularly in patients who are at risk of opioid addiction or have experienced adverse effects from opioid therapy[7].

Suzetrigine’s clinical development includes rigorous Phase 2 and Phase 3 randomized, double-blind, placebo- and active-controlled trials. In the NAVIGATE-1 and NAVIGATE-2 trials, patients undergoing abdominoplasty or bunionectomy received either suzetrigine, placebo, or hydrocodone/acetaminophen. Suzetrigine significantly reduced pain versus placebo over 48 hours with a faster onset of relief (median 2–4 hours vs. 8 hours for placebo). In pooled analyses of the double-blind studies (total *n* ≈ 874), the most common adverse events on suzetrigine were mild to moderate – pruritus, muscle spasms, CPK elevation, and rash – with no serious treatment-related events^[8,9]^.

In a separate Phase 2 trial for chronic lumbosacral radiculopathy, Suzetrigine met its own within-group endpoint (mean NPRS change – 2.02 at Week 12, *P* < 0.001 versus baseline) but placebo patients improved almost identically (–1.98). Because the study was not powered for between-group comparison, this numerical tie meant no statistically significant difference was observed versus placebo. In short, suzetrigine’s pain reduction did not outperform placebo. Importantly, suzetrigine was well tolerated in this chronic pain trial: there were no serious adverse events or discontinuations due to treatment[10].

These results suggest that suzetrigine’s efficacy is most pronounced in the acute postsurgical setting studied in NAVIGATE. Subgroup analyses from NAVIGATE hint at the breadth of its effect: for example, among patients with higher baseline pain, suzetrigine provided much faster relief than placebo. A limitation of the Phase 3 data is the trial demographics: only about 2% of abdominoplasty and 15% of bunionectomy participants were male, so extrapolating to male patients carries uncertainty. Nonetheless, the totality of evidence rigorous trial designs, statistically significant pain reductions versus placebo, and favorable comparisons with opioids in terms of side effects and abuse potential – supports suzetrigine’s role as an effective non-opioid analgesic for moderate to severe acute pain^[8,9]^.

However, the most common adverse reactions in study participants who received Suzetrigine were itching, muscle spasms, increased blood level of creatine phosphokinase, and rash. Suzetrigine is contraindicated for simultaneous use with strong CYP3A inhibitors[7]. Additionally, patients should avoid food or drink containing grapefruit while taking Suzetrigine. The long-term safety profile of suzetrigine remains to be fully established, and further clinical trials are expected to provide insight into its safety with prolonged use. Additionally, more data are needed to clearly define the quantitative relationship between *in vitro* pain signal inhibition and clinical efficacy. While suzetrigine has shown promise in treating acute pain, its effectiveness in chronic pain conditions particularly those involving central sensitization remains uncertain[6].

The application of Artificial intelligence such as AlphaFold offers transformative potential for protein structure prediction and novel drug target discovery remarkably[11]. AI can help find new treatment targets much faster, which is really beneficial for the development of Suzetrigine and other non-opioid painkillers. Furthermore, by analyzing detailed patient data such as genetic profiles, medical history, and types of pain, AI can formulate more personalized pain management plans that work better and cause fewer side effects[12]. In addition to this, AI can enhance the way clinical trials are designed and carried out by processing large datasets to identify the most important factors impacting drug safety and effectiveness.

By closely tracking prescription habits and patient behavior we can better spot drug abuse in real time. Incorporating AI into the study of Suzetrigine would not only introduce innovative approaches but also open up important paths for future research, particularly in accelerating drug development, improving pain management, enhancing clinical trial efficiency, and preventing drug misuse.

In conclusion, Suzetrigine presents a hopeful substitute for opioids in the treatment of acute pain. Its distinctive mechanism, absence of addictive properties, and reduced side effects make it a safer choice for individuals vulnerable to opioid dependence. Although further research is essential, the promising results from its clinical trials indicate potential for managing pain without the risks associated with opioids, providing optimism for a resolution to the persistent opioid crisis.

## Data Availability

Data sharing is not applicable to this article.
